# Case report: Systemic sclerosis during neoadjuvant therapy for breast cancer in a 59-year-old woman

**DOI:** 10.3389/fimmu.2024.1487508

**Published:** 2024-12-13

**Authors:** Siyu Liu, Xiaomei Xiao, Fangjing Yue, Cong Su, Yujun Tong, Weiyun Xu

**Affiliations:** ^1^ Department of Breast Surgery, the Affiated Hospital of South West Medical University, Luzhou, China; ^2^ Department of Emergency Medicine, Suining Central Hospital, Suining, China; ^3^ Department of Neurosurgery, Mianyang Central Hospital, School of Medicine, University of Electronic Science and Techology of China, Mianyang, China; ^4^ Department of Breast Center, Mianyang Central Hospital, School of Medicine, University of Electronic Science and Technology of China, Mianyang, China

**Keywords:** breast cancer, systemic sclerosis, pathogenesis, treatment, diagnosis

## Abstract

Systemic sclerosis (SSc) is an autoimmune connective tissue disease with skin fibrosis being the first and most common manifestation. Patients with SSc have a higher risk of developing malignant tumors than the general population. However, the sequence and underlying mechanisms linking SSc to malignancy remain controversial. This article presents the case of a 59-year-old woman who was diagnosed with SSc after developing skin fibrosis during neoadjuvant therapy for breast cancer. Despite aggressive antitumor treatments, including targeted therapy, SSc did not improve and progressed rapidly with increasing dermatofibrosis. Remarkably, the SSc entered remission following the cessation of antitumor therapy. Additionally, we reviewed the literature on SSc and malignant tumors, examined their relationship, and discussed key points regarding their identification and potential for adverse drug reactions.

## 1 Introduction

Systemic sclerosis (SSc) is an autoimmune connective tissue disorder characterized by cutaneous fibrosis and multi-organ involvement. The exact etiology and pathogenesis of SSc remain unclear, although it SSc is associated with endothelial cell dysfunction and abnormal fibroblast regulation, leading to skin and internal organ fibrosis. Patients with SSc have a higher risk of developing malignant tumors than the general population, with lung, breast, and hematologic cancers being the most common malignancies (1, 2). The connection between SSc and cancer is not fully understood. Some studies (3, 4) suggest that SSc may act as a paraneoplastic syndrome, while others propose (5) that both SSc and malignancies arise from shared immune dysfunction. Although breast cancer is frequently reported in patients with SSc, cases of rapidly progressing SSc during breast cancer treatment are rare. This article presents a case of SSc that rapidly advanced during breast cancer treatment.

## 2 Case description and diagnostic assessment

In October 2023, a 59-year-old female patient presented to the Breast Disease Center at Mianyang Central Hospital with a one-year history of a lump in her right breast. The key milestones in patient diagnosis and treatment are summarized in **Supplementary Table S1**, and SSc manifestations at different stages are detailed in **Supplementary Table S2** (the details of **Supplementary Tables S1** and **S2** are in the **Supplementary Material**). On examination, the patient’s vital signs were stable. Physical examination revealed an irregular mass in the right breast, measuring 12.5 cm × 8.0 cm, with indistinct borders. The skin over the areola displayed an orange-peel texture. The remaining skin color and elasticity, as well as range of motion in the limbs, were normal. The patient had no significant medical or family history, nor was she taking any medications.

Liver and kidney function tests, complete blood count, and other routine test results were within normal limits. Mammography, ultrasonography, and contrast-enhanced magnetic resonance imaging confirmed the presence of a malignant mass in the right breast with abnormally enlarged axillary lymph nodes. No distant metastases were detected in other imaging studies.

Core needle biopsy confirmed the diagnosis of invasive ductal carcinoma of the right breast with axillary lymph node involvement. Immunohistochemistry results further corroborated this diagnosis. The final diagnosis was right-sided invasive ductal carcinoma with ductal carcinoma *in situ* (cT4N2M0, Stage IIIb), classified as a luminal B HER-2 positive subtype. The patient underwent neoadjuvant therapy with six cycles of TcbHP comprising docetaxel, carboplatin, trastuzumab, and pertuzumab.

After the second chemotherapy cycle, the patient experienced itching, skin hardening, and hyperpigmentation in the upper outer quadrant of the right breast at the 11 o’clock position, covering 3.0 × 5.0 cm. Initially, this was hypothesized to be an adverse reaction to chemotherapy; therefore, no special treatment was provided and the patient continued with chemotherapy. However, as treatment progressed, the itching worsened, and the sclerosis and hyperpigmentation gradually spread from the breast to the neck and face. By the end of the fifth chemotherapy cycle, the patient exhibited skin hardening, itching, and hyperpigmentation of the face and both upper limbs. Suspecting chemotherapy-induced dermatitis, loratadine and mometasone furoate cream were prescribed for symptomatic relief, and the patient proceeded with the sixth chemotherapy cycle.

**Figure 1:**
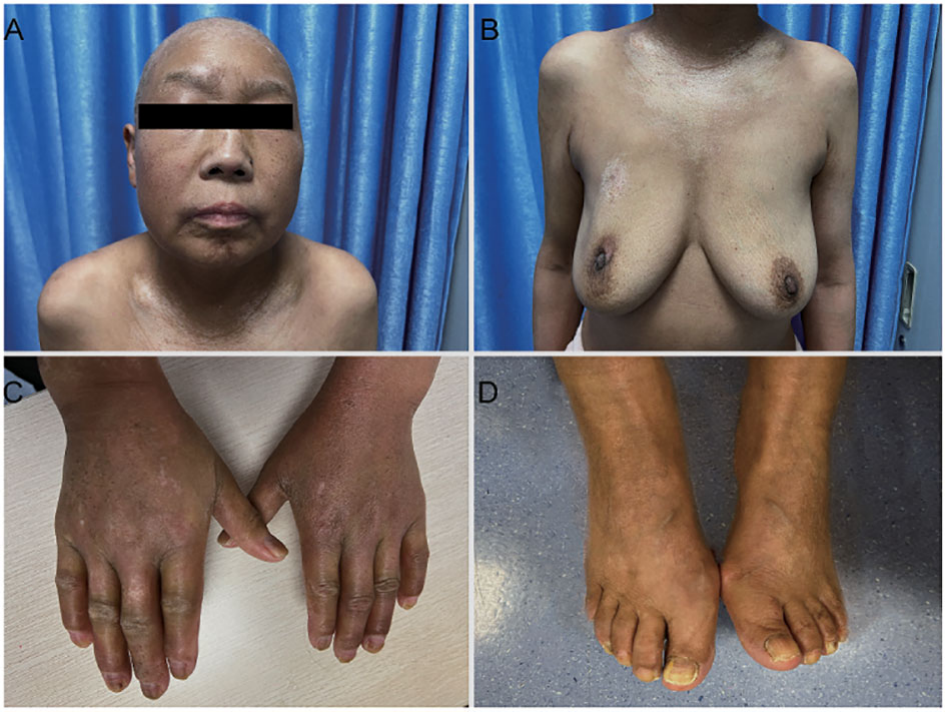
Skin manifestations at the time the patient was diagnosed with SSc. The skin of the limbs and face showed sclerosis, the chest and neck developed a distinctive “pepper-and-salt” **(A–D)**.

After completing chemotherapy, the patient developed pronounced skin sclerosis, primarily affecting the limbs and face. The skin was taut, swollen, and displayed non-pitting edema. Her fingers appeared puffy, with loss of skin folds and restricted range of motion. Her facial expression was stiff and mask-like. Hyperpigmentation intensified on the face, waist, and abdomen, whereas the skin on the chest and neck developed a distinctive “pepper-and-salt” appearance (**Figure 1**). Throughout the neoadjuvant therapy, dermatosclerosis and hyperpigmentation progressed from the breast to the head, neck, lower back, abdomen, and extremities. Medication such as loratadine and mometasone furoate cream failed to halt the progression of the condition. Given the worsening of symptoms, the possibility of comorbid skin or immune-related diseases was considered. Upon admission, routine autoantibody testing was performed to assess for a comorbid immune disorder, which showed as follow: C-reactive protein (CRP) levels of 0.25 ng/dL, anti-Streptolysin O (ASO) concentrations of 0.25 IU/mL, and rheumatoid factor (RF) levels of <20 IU/mL. The patient tested negative for anti-cyclic citrullinated peptide antibodies (anti-CCP) were negative. Considering the possibility of an underlying immune disorder, an indirect immunofluorescence assay (IIFA) revealed the presence of antinuclear antibodies (ANA) with both a speckled pattern (AC-4/5) at a titer of 1:3200 and a dense fine speckled pattern (AC-2) at a titer of 1:320. Further analysis identified anti-Scl-70 antibody positivity. The patient was subsequently diagnosed with SSc and the modified Rodnan skin score (mRSS) was 19.

Glucocorticoids may be considered for patients with significant skin swelling to control inflammation and alleviate edema. However, the use of rituximab (RTX), tocilizumab (TCZ), and glucocorticoids during the perioperative period may increase the risks of poor incision healing, flap necrosis, and infection (6–9). Given these concerns, hydroxychloroquine, which has immunomodulatory properties and can inhibit abnormal immune responses without compromising normal immune function, was chosen as an alternative. Additionally, due to the patient’s limited financial resources to support the cost of TCZ and RTX treatments, more affordable treatment options were selected. Therefore, the patient was treated symptomatically with hydroxychloroquine (0.2 g bid), candesartan (8 mg qd), and beclomethasone (40 µg qd). After one week of treatment, the patient showed reduced skin tightness and improved mobility of their extremities. Following a multidisciplinary treatment discussion, we decided to proceed with modified radical mastectomy. Intraoperatively, significant skin fibrosis complicated the procedure, making breast flap dissection difficult. The area of the skin first affected by sclerosis, near the breast cancer lesion, was excised, and a flap graft was performed.

**Figure 2:**
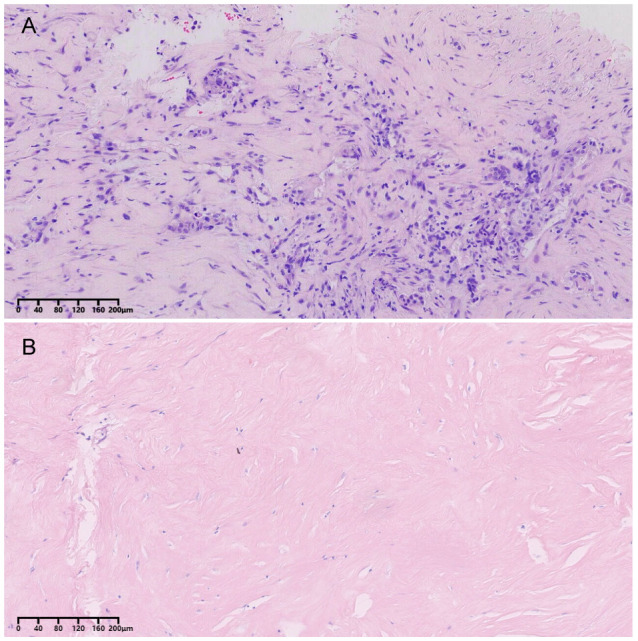
Postoperative breast cancer tissue skin pathology results. **(A)** Hematoxylin and eosin (HE) stain of breast cancer tissue (magnification ×100); **(B)** HE stain of sclerotic skin (magnification ×100).

Postoperative skin biopsy revealed epidermal keratinization with extensive dermal collagenization and perivascular chronic inflammatory cell infiltration, consistent with scleroderma (**Figure 2**). The patient was discharged one week later with no signs of flap necrosis or effusion, and the incision healed well.

**Figure 3:**
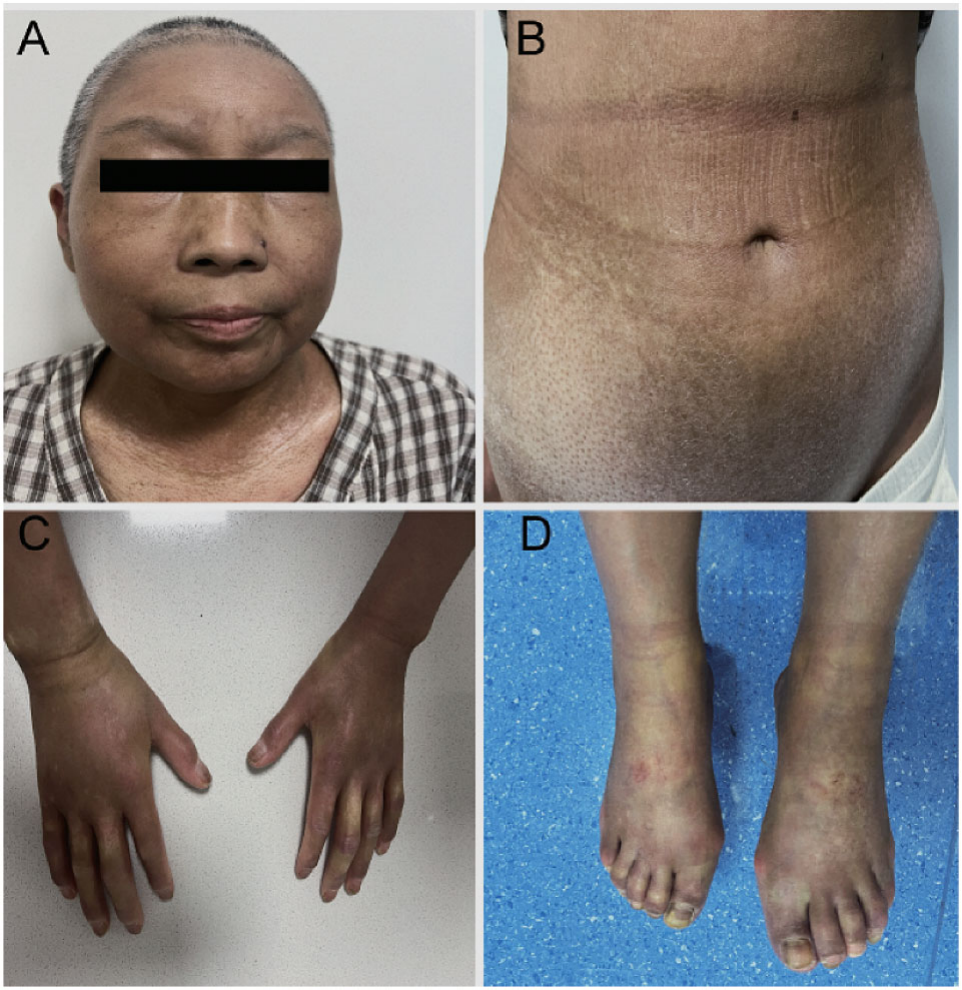
Skin manifestations following the rapid progression of systemic sclerosis, Generalized skin sclerosis, Raynaud’s phenomenon, swollen knuckles, and limited mobility **(A–D)**.

One month later, the patient continued targeted therapy with trastuzumab and pertuzumab. After completing two cycles, sclerosis of the skin on the patient’s face, limbs, waist, and abdomen worsened, accompanied by generalized hyperpigmentation and pruritus. The hard swelling became more pronounced, rendering the skin impossible to pinch or twist. Shallow ulcers appeared on the dorsum of the patient’s foot, and their knuckles became swollen, painful, and stiff, limiting flexion and extension. Raynaud’s phenomenon was also prominent. Extensive fibrosis in the extremities led to unsteady walking and bilateral knee flexion was limited to > 90°, and an mRSS of 44 (**Figure 3**). However, the patient did not experience difficulty with breathing or swallowing, nor were there any signs of gastrointestinal involvement such as gastroesophageal reflux, indigestion, or diarrhea.

Chest computed tomography (CT) revealed interstitial fibrotic changes in both lungs, and cardiac ultrasonography suggested mild pulmonary hypertension. Liver and kidney functions remained normal. Given the progression of SSc, it was unclear whether this was related to antitumor drug treatment, and targeted therapy was discontinued. Given the patient’s significant swelling and high inflammatory response, yet mild internal organ involvement, we opted to use glucocorticoids for inflammation control. The main clinical manifestation in this patient was severe, diffuse skin fibrosis. According to the 2023 EULAR guidelines, mycophenolate mofetil is recommended as a first-line treatment for skin fibrosis in SSc (10). Both thalidomide and colchicine exhibit antifibrotic properties (11, 12). Due to the progression of SSc, we discontinued antitumor therapy. Given that both thalidomide and colchicine exhibit antitumor effects (13–16), we combined them with low-dose mycophenolate mofetil to enhance safety in the oncological setting while also control the progression of SSc. At the same time, our treatment also adhered to the Guidelines for the Diagnosis and Treatment of Systemic Sclerosis issued by the Rheumatology Branch of the Chinese Medical Association (17). The patient’s immunosuppressive regimen was adjusted to include prednisone acetate (100mg qd for short term use, then adjusted to 15mg qd), colchicine (0.5 mg qd), thalidomide (50 mg qn), hydroxychloroquine (0.2g bid), and mycophenolate mofetil (0.5g bid) to aggressively manage SSc and prevent further progression. The echocardiographically suggested pulmonary hypertension did not worsen or change from the time of admission, and influenced by the patient’s financial constraints, so we did not further examine the right heart catheterization or perform drug intervention. Antitumor therapy was reconsidered once the patient’s condition had stabilized.

Due to the severe dermal fibrosis and the recognition of SSc as a relative contraindication to radiotherapy according to the National Comprehensive Cancer Network (NCCN) guidelines, postoperative radiation therapy was deferred to avoid exacerbating the patient’s condition. The patient was advised to undergo regular monitoring, including chest CT, cardiac ultrasound, and liver and renal function tests, to detect early signs of organ damage and assess the appropriate timing for resuming antitumor therapy.

After three months of follow-up, the patient showed improvement in SSc symptoms and limb mobility after discontinuing anticancer therapy, and the patient continues to experience skin sclerosis and pigmentation on the neck, face, limbs, waist, and abdomen. The skin had become more flexible, with significant reductions in tightness and itching. Partial healing of the foot ulcer was observed. Knuckle swelling subsided, and the range of motion improved. Pain, swelling, and stiffness were markedly reduced. No evidence of breast cancer recurrence or metastasis was detected at this time.

## 3 Discussion

The association between breast cancer and SSc remains unclear. While the sequence of disease occurrence is documented, the mechanisms linking these conditions remain debated. One characteristic of SSc is the imbalance between pro-angiogenic and anti-angiogenic mediators, along with the dysfunction of endothelial progenitor cells. Endothelial cell activation is crucial for angiogenesis in breast tumors and is a key process in cancer development, invasion, progression, and metastasis (18). Overexpression of vascular endothelial growth factor in SSc may promote the development of breast cancer, suggesting that endothelial cell activation could be a pathophysiological link between SSc and breast cancer. Similarly, platelet-derived growth factor and fibroblast growth factor are elevated in patients with SSc and may contribute to breast cancer development (5). Certain antibodies associated with SSc, such as anti-RNA polymerase III and anti-topoisomerase I, may interact with cellular DNA, altering its structure and potentially promoting cancer development (1, 19). Cohort studies have revealed that anti-RNA polymerase III and anti-topoisomerase I antibodies are risk factors for lung cancer (20–22); however, their roles in breast cancer remain controversial.

There is a complex dual relationship between systemic sclerosis (SSc) and malignancy, involving the immune system, genetic and epigenetic changes, environmental factors, and oxidative stress. Autoimmunity in SSc may be triggered by antigenic mutations in tumor cells (2, 23, 24). Maria ATJ et al. (23) reviewed extensive literature, concluding that in patients with specific autoantibodies, such as anti-RNA polymerase III, SSc may function as a paraneoplastic syndrome linked to anticancer immune responses. Similarly, Shah AA et al. (25) found a close temporal relationship between the onset of scleroderma and the diagnosis of malignancy in patients with cancer-associated scleroderma who produce anti-RNA polymerase autoantibodies. They reported that RNA polymerase III expression was significantly elevated in tumors of patients with anti-RNA polymerase antibodies after comparing clinical and demographic features across autoantibody classes. This finding underscores a strong association between tumor antigen expression and scleroderma autoantibodies, highlighting RNA polymerase III as a potential target of tumor-associated antigens. Together, these findings suggest that, in some patients, scleroderma may be a by-product of the anti-tumor immune response. Similar conclusions were drawn from larger cohort studies (22, 26, 27).

Joseph CG, et al. (28) reported that POLR3A mutations, each causing an amino acid change, are somatic alterations. These mutations were identified in tumors of eight patients with anti-RNA polymerase III antibody-positive cancers and eight with anti-topoisomerase I-positive cancers. The absence of these mutations in tumors from patients with other antibody profiles suggests that mutated RNA polymerase III proteins may act as immunogens, initiating anti-RNA polymerase III antibody responses. The emergence of scleroderma in the context of malignancy may be the “price” of eliminating cancer as a paraneoplastic disease.

Derk (3) conducted a case-control study comparing patients diagnosed with breast cancer both before and after the onset of SSc. The study suggests that SSc development post-breast cancer is more likely a paraneoplastic syndrome than an autoimmune response. Notably, ANA was absent in patients developing SSc after breast cancer but present in those with breast cancer onset after SSc. In this case, the patient tested positive for ANA, with a high antibody titer. Lu et al. (4) reached a similar conclusion, observing that SSc occurring after breast cancer often improved with antitumor therapy and worsened with recurrence. In the case of this patient, however, SSc did not resolve with tumor treatment. Additionally, the patient was not further examined for other rare antibodies or subjected to genetic analysis. As a result, it remains difficult to determine whether this patient’s SSc is a paraneoplastic syndrome or a primary autoimmune disease.

There are various explanations for the occurrence of SSc after tumor treatment; however, it is important to consider that breast cancer treatment may also contribute to the onset of SSc. Taxanes are first-line treatments for breast cancer, and skin and subcutaneous changes caused by taxanes are generally mild to moderate and reversible, often manifesting as rash and rarely leading to functional impairment. Scleroderma-like changes are a rare adverse effects of taxanes. Although the mechanisms by which taxanes induce SSc remain unclear, two possible pathways have been proposed. First, taxanes may induce the activation of fibroblasts, which secrete proteoglycans that interact with other cells, promoting their proliferation, migration, adhesion, and the formation of extracellular matrix, leading to skin thickening. Second, taxanes may induce immune system stimulation and inflammatory response. Inflammatory cells typically accumulate at the dermal-adipose junction, while inflammatory factors produced by these cells are elevated in the serum of patients with SSc, suggesting that they may play a role in SSc pathogenesis (29–32).

Previous case reports (31, 33, 34) indicate that taxane-induced skin hardening often resolves quickly after drug discontinuation. Initially, the observed skin changes were attributed to adverse reactions to taxanes. However, the patient’s skin sclerosis persisted after chemotherapy and worsened with subsequent targeted therapy, suggesting an additional underlying condition. There are few reports on whether there is an association between anti-HER2 therapy and SSc; therefore, more research is still needed.

Early manifestations of SSc typically include Raynaud’s phenomenon, swelling of extremities, facial swelling, and progressive skin changes characterized by swelling, hardening, and atrophy. Skin fibrosis typically progresses from the extremities toward the trunk. However, in this case, the patient initially presented with localized skin sclerosis and pigmentation in the breast, which later spread outward to other body regions. This atypical progression delayed the diagnosis of SSc in this patient.

Early immunotherapy can help control the rapid progression of skin fibrosis in patients with SSc (35–37). In this case, delayed diagnosis and treatment occurred after extensive fibrosis had developed in the limbs and trunk, which likely accelerating disease progression. Autoantibody detection is essential for the early diagnosis and prognosis of SSc. Highly specific autoantibodies, including anti-topoisomerase I, anti-centromere, and anti-RNA polymerase III, are associated with rapid skin progression in SSc patients. Early-onset extensive or rapidly progressing fibrosis generally indicates a poor prognosis. Strong anti-Scl-70 positivity in this patient suggested aggressive disease progression and a poor prognosis. Autoantibody testing in patients with skin changes during chemotherapy can aid in differentiating drug reactions from early SSc. Raynaud’s phenomenon, extremity swelling, and hyperpigmentation are shared features of chemotherapeutic side effects and early SSc. Thus, monitoring these overlapping symptoms is critical for timely diagnosis and management.

Paclitaxel, platinum, and other chemotherapeutic agents have been reported to induce Raynaud’s phenomenon (38, 39). Chemotherapy-induced Raynaud’s phenomenon usually manifests within 3–6 months of treatment and is dose-dependent. Differentiating chemotherapy-induced Raynaud’s phenomenon from the early onset of an immune-mediated disease is crucial in chemotherapy patients. Timely intervention can prevent progression to severe complications, including ulcers, necrosis, and gangrene.

Paclitaxel analogs often cause reversible peripheral edema, which can occur at varying times. Research suggests a correlation between edema and cumulative drug dosage; however, swelling usually resolves upon discontinuation. In contrast, the initial phase of SSc skin lesions involves swelling, often accompanied by pruritus and pain, resembling chemotherapy-induced side effects. The diagnosis of autoimmune diseases, including SSc, depends heavily on detecting specific autoantibodies (40). The European Scleroderma Trial and Research Group (EUSTAR) has introduced the concept of very early diagnosis of systemic sclerosis (VEDOSS) (41). In patients presenting with Raynaud’s phenomenon, puffy fingers, and positive antinuclear antibodies, early screening for SSc-related antibodies and monitoring for visceral involvement is crucial. Early identification of drug-induced adverse reactions in SSc patients can be facilitated through autoantibody testing and monitoring visceral organ function.

Cytotoxic chemotherapeutic agents can induce hyperpigmentation with varying manifestations, often appearing within weeks of treatment initiation. Hyperpigmentation usually resolves 6–12 months after discontinuing chemotherapy but may persist in some cases (42, 43). SSc can also cause hyperpigmentation or depigmentation, resulting in characteristic salt-and-pepper skin changes (44, 45). This salt-and-pepper appearance is a hallmark of SSc and may represent one of the earliest cutaneous manifestations of the disease. It commonly appears on the scalp, neck, thoracic dorsum, forearms, and dorsal fingers, typically distributed bilaterally and symmetrically. Chemotherapy-induced hyperpigmentation is usually systemic and drug-specific. Salt-and-pepper skin may be the earliest or sole cutaneous sign of SSc, aiding early diagnosis. Recognizing this distinctive sign is vital for timely SSc diagnosis.

This case highlights a dilemma: continuing antitumor therapy risks worsening SSc and causing life-threatening organ damage, while suspending therapy risks breast cancer progression or recurrence. The relationship between targeted drugs and SSc remains unclear. Given prior neoadjuvant therapy and surgery, we chose to halt further antitumor therapy and prioritize aggressive SSc treatment. SSc remission and breast cancer stabilization suggest a novel approach to managing such conflicting treatment strategies. We opted to discontinue intensive antitumor therapy after initial effective treatment, while actively managing the patient’s immune disorders to maintain a relative balance between the two diseases and prevent further exacerbation.

In this case, the patient exhibited severe skin fibrosis affecting nearly the entire skin surface; however, the internal organs were rarely involved and did not deteriorate with worsening skin lesions. This discrepancy complicates understanding the etiology of SSc and its progression. The relationship between malignant tumors and SSc remains uncertain, and further studies are required to elucidate the pathophysiological connections between these two conditions. The differences observed between this case and previous reports may provide new insights into this complex relationship.

## Data Availability

The original contributions presented in the study are included in the article/**Supplementary Material**. Further inquiries can be directed to the corresponding authors.
