# Supplementary material for: Case report: Systemic sclerosis during neoadjuvant therapy for breast cancer in a 59-year-old woman
Source: Front Immunol. 2024 Dec 13;15:1487508. doi: 10.3389/fimmu.2024.1487508 (PMC11671355; doi:10.3389/fimmu.2024.1487508)
Supplement: Supplementary file 1 [file Table1.docx]

Supplementary Material

TABLE 1 A timeline of key points in the patient’s diagnosis and treatment.

| Date | Symptom | Test/Diagnosis | Treatment |
| --- | --- | --- | --- |
| October 2023 | A lump was found in right breast | Right invasive ductal carcinoma with ductal carcinoma (cT4N2M0 IIIb), Luminal B-like (HER-2 positive) | Proposed a 6-cycle TcbHP regimen chemotherapy (“docetaxel + carboplatin + trastuzumab + patuximab” |
| November 2023 (2nd cycle of chemotherapy) | The skin in the upper outer quadrant of the right breast (at the 11 o'clock position) became itchy, hardened, and hyperpigmented, with a range of about 3.0 cm x 5.0 cm | Adverse effects of chemotherapy | No treatment was given |
| January 2024 (5th cycle of chemotherapy) | Dermatosclerosis of both upper extremities and facial hyperpigmentation | Adverse reactions of hyperpigmentation and dermatitis caused by chemotherapy. | Loratadine and mometasone furoate cream |
| March 2024 | The skin of the limbs and face showed sclerosis, non-pitting edema (non-pitting hard swelling), and a tight, swollen feeling. | Systemic sclerosis (SSc). | Hydroxychloroquine (0.2g bid), candesartan (8mg qd), and beclomethasone (40ug qd) |
| March 2024 | Reduced skin tightness and improved mobility of the extremities | Right invasive ductal carcinoma (ycT3N1M0 IIIA) | Modified radical mastectomy |
| April 2024 | Generalized skin sclerosis, Raynaud's phenomenon, swollen knuckles, and limited mobility after the eighth postoperative targeted therapy session. SSc progressed observed. |  | Suspension of anti-tumor therapy. Initiated treatment with prednisone acetate (100mg qd for short term use, then adjusted to 15mg qd), colchicine (0.5mg qd), thalidomide (50mg qn)， hydroxychloroquine (0.2g bid), and Mycophenolate mofetil (0.5g bid) |
| July 2024 | Improved limb mobility, reduced skin tightness, and decreased knuckle swelling compared to previous evaluations. |  |  |

Table 2 SSc manifestations of patients in different periods

| Stage | Time | Skin manifestations | Auxiliary examination |
| --- | --- | --- | --- |
| Initial period | November 2023 - March 2024 | Initially, the right breast skin pruritus, hardening and pigmentation (3cm*5cm). After that, the limbs, neck and face, waist and abdomen gradually appear skin itching, hardening and pigmentation, tight bondage is obvious, the skin can still twist up. The fingers became puffy, their skin folds disappeared, their range of motion was limited. | Chest CT：interstitial fibrotic changes in both lungs  Cardiac ultrasound：mild pulmonary hypertension  Liver and kidney function：normal |
| Remission periods | March 2024 | The skin tightness and swelling of the fingers are reduced, and the mobility of the limbs is increased. | Chest CT：interstitial fibrotic changes in both lungs  Liver and kidney function：normal |
| Aggravating period | April 2024 | The range of skin sclerosis of neck, face, limbs and waist and abdomen is enlarged, pigmentation is aggravated, and the skin cannot be twisted up. Superficial ulcers appear on the skin of the foot. The finger joints are swollen and stiff, and the flexion and extension are not easy, Raynaud's phenomenon is obvious. | Cardiac ultrasound：mild pulmonary hypertension  Liver and kidney function：normal |
| Remission period | July 2024 | There are still skin sclerosis and pigmentation in the neck, face, limbs, waist and abdomen, but the skin can twist up and the skin tightness is reduced. The skin ulcer on the foot has healed. The swelling of the finger joints is reduced and the range of motion is increased. | Liver and kidney function：normal |
